# Knowledge mapping analysis of ground glass nodules: a bibliometric analysis from 2013 to 2023

**DOI:** 10.3389/fonc.2024.1469354

**Published:** 2024-09-24

**Authors:** Linfeng Wang, Ayidana Maolan, Yue Luo, Yue Li, Rui Liu

**Affiliations:** Department of Oncology, Guang’anmen Hospital, China Academy of Chinese Medical Sciences, Beijing, China

**Keywords:** ground glass nodule, lung cancer, bibliometrics, citespace, VOSviewer

## Abstract

**Background:**

In recent years, the widespread use of computed tomography (CT) in early lung cancer screening has led to an increase in the detection rate of lung ground glass nodules (GGNs). The persistence of GGNs, which may indicate early lung adenocarcinoma, has been a focus of attention for scholars in the field of lung cancer prevention and treatment in recent years. Despite the rapid development of research into GGNs, there is a lack of intuitive content and trend analyses in this field, as well as a lack of detailed elaboration on possible research hotspots. The objective of this study was to conduct a comprehensive analysis of the knowledge structure and research hotspots of lung ground glass nodules over the past decade, employing bibliometric methods.

**Method:**

The Web of Science Core Collection (WoSCC) database was searched for relevant ground-glass lung nodule literature published from 2013-2023. Bibliometric analyses were performed using VOSviewer, CiteSpace, and the R package “bibliometrix”.

**Results:**

A total of 2,218 articles from 75 countries and 2,274 institutions were included in this study. The number of publications related to GGNs has been high in recent years. The United States has led in GGNs-related research. Radiology has one of the highest visibilities as a selected journal and co-cited journal. Jin Mo Goo has published the most articles. Travis WD has been cited the most frequently. The main topics of research in this field are Lung Cancer, CT, and Deep Learning, which have been identified as long-term research hotspots. The GGNs-related marker is a major research trend in this field.

**Conclusion:**

This study represents the inaugural bibliometric analysis of applied research on ground-glass lung nodules utilizing three established bibliometric software. The bibliometric analysis of this study elucidates the prevailing research themes and trends in the field of GGNs over the past decade. It also furnishes pertinent recommendations for researchers to provide objective descriptions and comprehensive guidance for future related research.

## 1 Introduction

Lung cancer remains one of the most prevalent and lethal malignant neoplastic diseases worldwide, with an estimated 1.8 million lung cancer deaths globally in 2022, accounting for 18.7% of all cancer-related deaths (1). Consequently, the prevention and treatment of lung cancer remains an important focus of medical research. Early diagnosis and treatment of lung cancer are important for prolonging the survival time and improving the prognosis of lung cancer patients (2). Studies have shown that persistent ground-glass nodules (GGNs) on imaging may be an important sign of early lung cancer and may play an important role in the early detection of lung cancer (3). A study of computed tomography (CT) screening in the Chinese population showed that approximately 95% of patients ultimately diagnosed with lung cancer had GGNs on imaging (4). Based on the density characteristics of GGNs on imaging, GGNs can be classified into pure ground glass nodule (pGGN) and mixed ground glass nodule (mGGN) with a solid component, which is more malignant than pGGN, and the presence and growth of a solid component is one of the features of the progression of precancerous lung lesions (5). Pathologically, the different degrees of progression of GGNs correspond to atypical adenomatous hyperplasia (AAH), adenocarcinoma *in situ* (AIS), minimally invasive adenocarcinoma (MIA), and invasive adenocarcinoma (IA) in progressive stages (6).

In the past decade or so, the advent of low-dose CT scanning has enabled a significant increase in the detection of small pulmonary nodules, commonly referred to as ground-glass nodules. Studies on screening populations in different countries and regions have shown that the detection rate of GGNs ranges from 0.42% to 22.9% (4, 7–11). However, GGNs have a high degree of heterogeneity, and detection of GGNs does not necessarily mean that early-stage lung cancers are found, and the detection rate of early-stage lung cancers is only about 5% of the screened population (3). Clinical diagnosis, treatment strategies, and timing of intervention for GGNs remain controversial, and follow-up observation after the first detection has become the main treatment option for patients with GGNs (6). The long follow-up time and premature pre-cancer diagnosis not only aggravate the anxiety of patients but also bring a heavy medical burden with frequent imaging screening and premature surgery, so how to accurately diagnose and treat GGNs to prevent and treat lung cancer has become a key issue in medical research, and related multidisciplinary studies and clinical trials are gradually being carried out and have received widespread attention. GGNs have become a popular topic of discussion among medical researchers of respiratory and oncological diseases.

Faced with a large volume of published literature, if we do not adopt appropriate reading and analysis strategies, we can become overwhelmed by the volume of literature and thus fail to grasp the focus of the field. Although a literature review can provide a useful overview of the current state of research in a given field, it is inherently subjective and therefore not always an accurate representation of the content of the research. Bibliometrics is a scientific, quantitative approach to literature analysis that employs a powerful statistical method to analyze the relevant characteristics of published publications. This enables the identification of bibliometric relationships between authors, organizations, countries, and references in a given field of study (12). The application of bibliometrics to the field of medical research enables a rapid understanding of the development trends within the discipline, the identification of current research hotspots, and the formulation of future research directions. This information can be used by researchers to inform their research plans, thereby accelerating the scientific research process. Consequently, bibliometrics is a suitable methodology for identifying research hotspots in GGNs. Common bibliometric tools include VOSviewer, CiteSpace, and the R package “bibliometrix”, which have been extensively employed in the medical field.

Li (13) summarized the bibliometric analysis of lung nodule-related bibliometrics from 1970 to 2020. It concluded that “Deep Learning” and “Artificial Intelligence” were the hot trends at that time. However, the study population was not specifically identified as ground-glass lung nodules. Considering the inert progression characteristics of GGNs, the diagnosis, treatment, and management of GGNs are also different from solid nodules, and the research on GGNs has made great progress in the last 3 years, the relevant hotspots and development trends have changed. However, no scholarly quantitative and qualitative studies of GGNs have been conducted in recent years. This study presents an analysis of GGNs-related research from January 1, 2013, to December 31, 2023. The aim is to comprehensively understand the evolving research trends and future development prospects in this field, visually delineate the scope of this domain for scholars, and facilitate the formulation of future research plans.

## 2 Materials and methods

### 2.1 Search strategy

**Figure 1:**
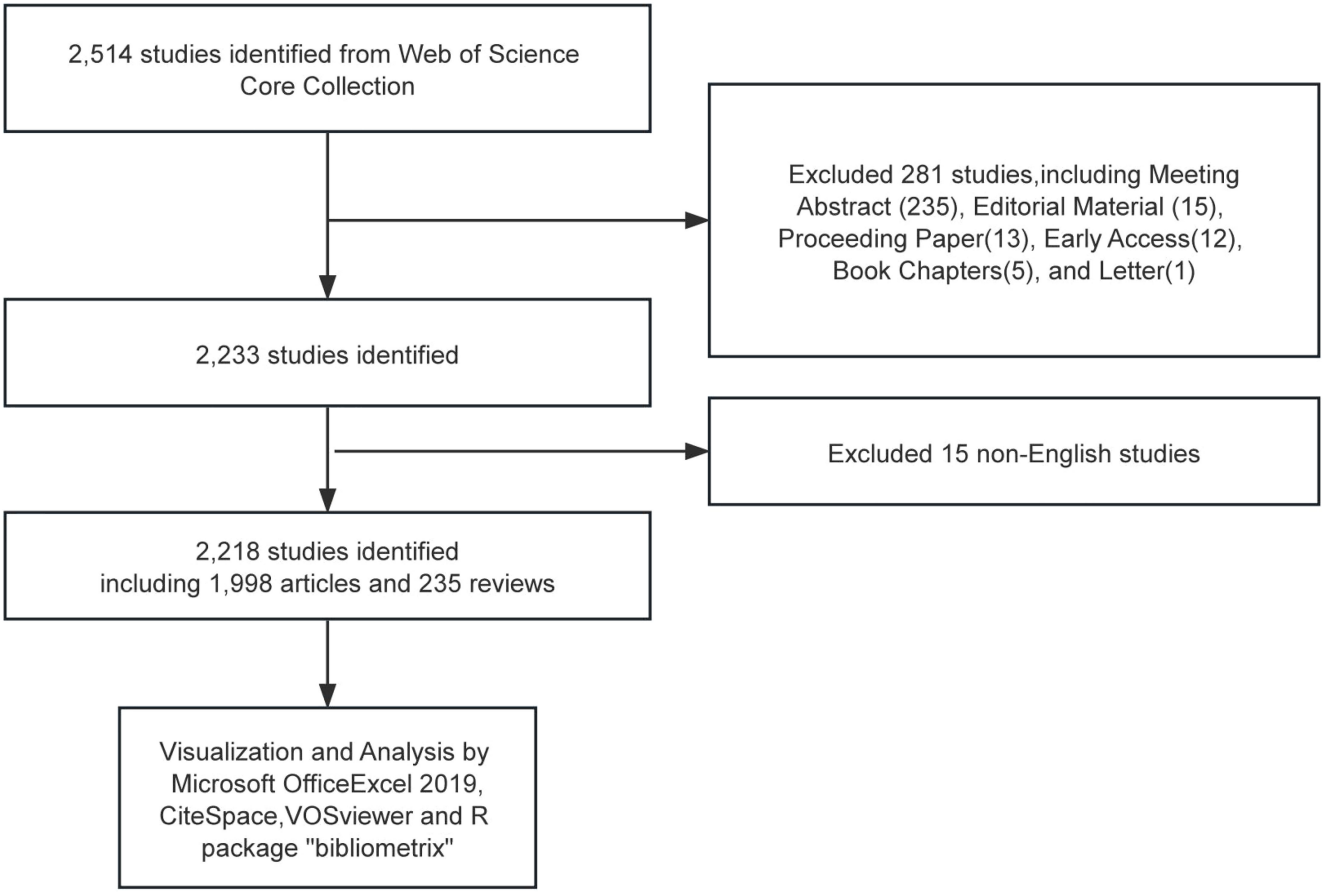
Publications screening flowchart.

We conducted a literature search of the Web of Science Core Collection (WoSCC) database, which includes the Science Citation Index (SCI), the Social Science Citation Index (SSCI), and several other databases with high impact and high authority. The time frame is limited to 1 January 2013 to 31 December 2023. Search strategy:((TS=(Solitary Ground Glass Opacity) OR TS=(Subsolid pulmonary nodule) OR TS=(Ground-glass nodule)) NOT TS=(Rheumatoid)) AND LA=(English), and the type of documents is set to “articles “and “review”. The procedure for data retrieval and collection is shown in **Figure 1**.

### 2.2 Visualized analysis

VOSviewer (version 1.6.20) is a programme that can be used to construct and view bibliometric maps and to extract key information from a large number of publications, often used to construct author or journal co-citation relationships and keyword co-occurrence relationships. In this paper, the software was used to analyze the relationships between authors, countries, institutions, journals, references, and keywords. In the map produced by VOSviewer, a node represents an item such as country, institution, journal, and author. The size and color of the nodes indicate the number and classification of these items, respectively. The distance between two nodes indicates the affinity between the two nodes; if the affinity is stronger, the distance is shorter, and if the affinity is weaker, the distance is further away, reflecting the degree of collaboration or co-citation of the projects (14). CiteSpace (version 6.2) is another application that supports visual exploration using knowledge mapping in bibliographic databases. In this paper, we use CiteSpace software to show trends in the annual number of publications related to GGNs research and analyze references using Citation Bursts. The R package “bibliometrix” (version 4.0.0) (https://www.bibliometrix.org) assisted in the thematic evolution analysis and the construction of a global publication distribution network for GGNs research. Journal impact factors were obtained from Journal Citation Reports 2023.

The full record of data obtained from the search was exported with cited references. After reasonable data cleaning, such as removing outliers, the files were imported into the analysis software to analyze the current status, hotspots, and problems of GGNs research.

## 3 Results

### 3.1 Quantitative analysis of publication

**Figure 2:**
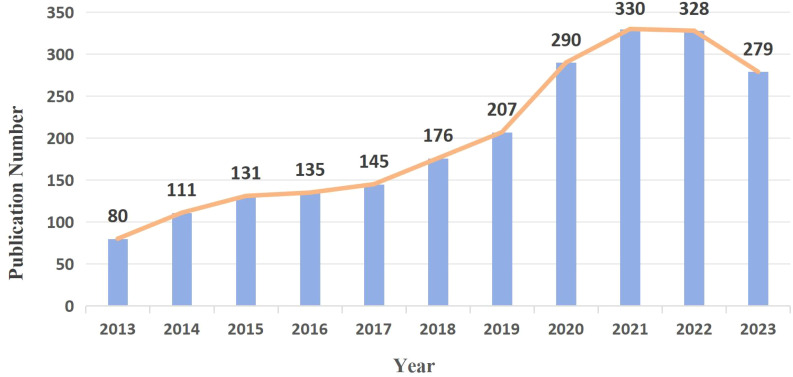
Annual output of research of the GGNs.

According to the search terms, 2,218 GGNs-related articles were retrieved from WoSCC. The exact number and trends of publications per year from 2013 to 2023 are shown in **Figure 2**. Over the past decade, GGNs research in general has been on a gradual upward trend, and the number of GGNs-related publications has been consistently high, indicating a long-term interest in the topic, with a significant increase in the total number of papers in 2020. A total of 328 studies were published in 2022, the highest number of studies published in the past decade.

### 3.2 Authors and co-cited authors analysis

**Figure 3:**
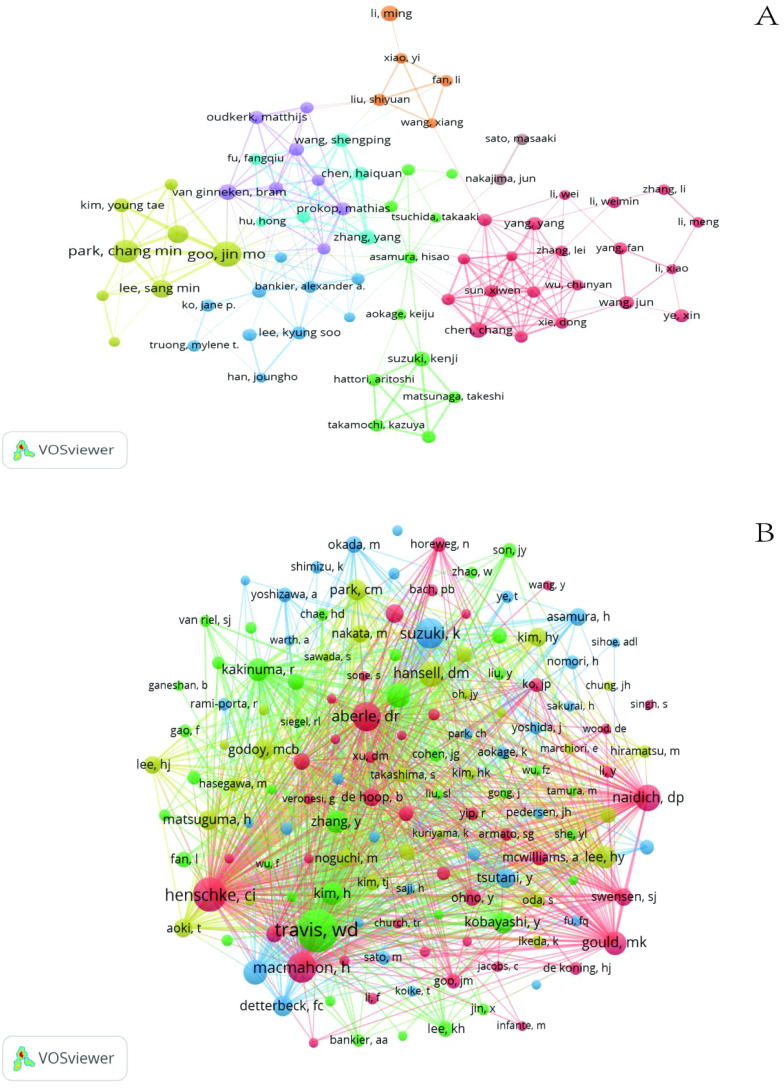
The visualization of authors **(A)** and co-cited Authors **(B)** on research of GGN.

A total of 10,910 authors published GGNs-related articles, and we built a collaborative network based on authors with 10 or more publications (**Figure 3**).

The collaboration mapping shows that most of the authors show a cluster-like distribution, with less connectivity between different clusters, as shown in **Figure 3A**. Among the top 10 authors (**Table 1**), Jin Mo Goo has the largest node representing his most relevant publications, with a total of 58 GGNs-related articles. In addition, we observe close collaboration between multiple authors. For example, Jin Mo Goo, who has the largest number of publications, has active collaborations with Chang Min Park.

**Table 1:** Top 10 authors and co-cited authors on research of application of GGN.

Rank	Authors	Count	Co-Cited Authors	Citations
1	Jin Mo Goo	58	Travis WD	960
2	Chang Min Park	48	Henschke CI	610
3	Lee Sang Min	31	Suzuki K	499
4	Hyung Jin Kim,	31	Macmahon H	492
5	van Ginneken B	23	Aberle DR	461
6	Li Ming	22	Naidich DP	380
7	Suzuki K	20	Hansell DM	355
8	Chen Chang	20	Hattori, A	333
9	Zhang Yang	20	Lee SM	326
10	Lv FJ	19	Gould MK	292

When multiple authors are cited together in one or more works, they are collectively referred to as co-cited authors. The analysis of co-cited authors showed that there were 21,037 co-cited authors, and the author with the highest number of co-citations was Travis WD (n=960), followed by Henschke CI (n=610) and Suzuki K (n=499) (**Table 2**). Authors with a minimum co-citation count of 50 were filtered to draw a co-citation network diagram (**Figure 3B**). As shown in **Figure 3B**, there is also an active collaboration between different co-cited authors, for example, Travis WD is closely related to Lee SM, Detterbeck FC, and Henschke CI is closely related to Naidich DP, Yankelevitz, Kim HY, and Zwirewich, CV.

**Table 2:** Top 10 journals and co-cited journals for research of GGNs.

Rank	Journal	Counts	IF	JCR	Co-cited Journal	Co-citation	IF	JCR
1	Journal of thoracic disease	100	2.5	Q4	Radiology	6,278	19.7	Q1
2	European radiology	93	5.9	Q1	Journal of Thoracic Oncology	3,657	20.4	Q1
3	Frontiers in oncology	65	4.7	Q2	American Journal of Roentgenology	2,898	5.0	Q1
4	Thoracic cancer	49	2.9	Q3	Chest	2,796	9.6	Q1
5	European journal of radiology	46	3.3	Q2	Annals of Thoracic Surgery	2,573	4.6	Q1
6	British journal of radiology	44	2.8	Q3	European Radiology	2,285	5.9	Q1
7	Medicine	43	1.6	Q3	Thoracic And Cardiovascular Surgeon	1,853	1.5	Q3
8	Radiology	40	19.7	Q1	Lung Cancer	1,675	5.3	Q2
9	Annals of thoracic surgery	37	4.6	Q1	New England Journal of Medicine	1,460	115.7	Q1
10	American journal of roentgenology	35	6.5	Q1	Journal of Thoracic Disease	1,066	2.5	Q4

IF, Impact Factor; JCR, Journal Citation Reports; Q, Quartile in Category.

### 3.3 Country analysis

**Figure 4:**
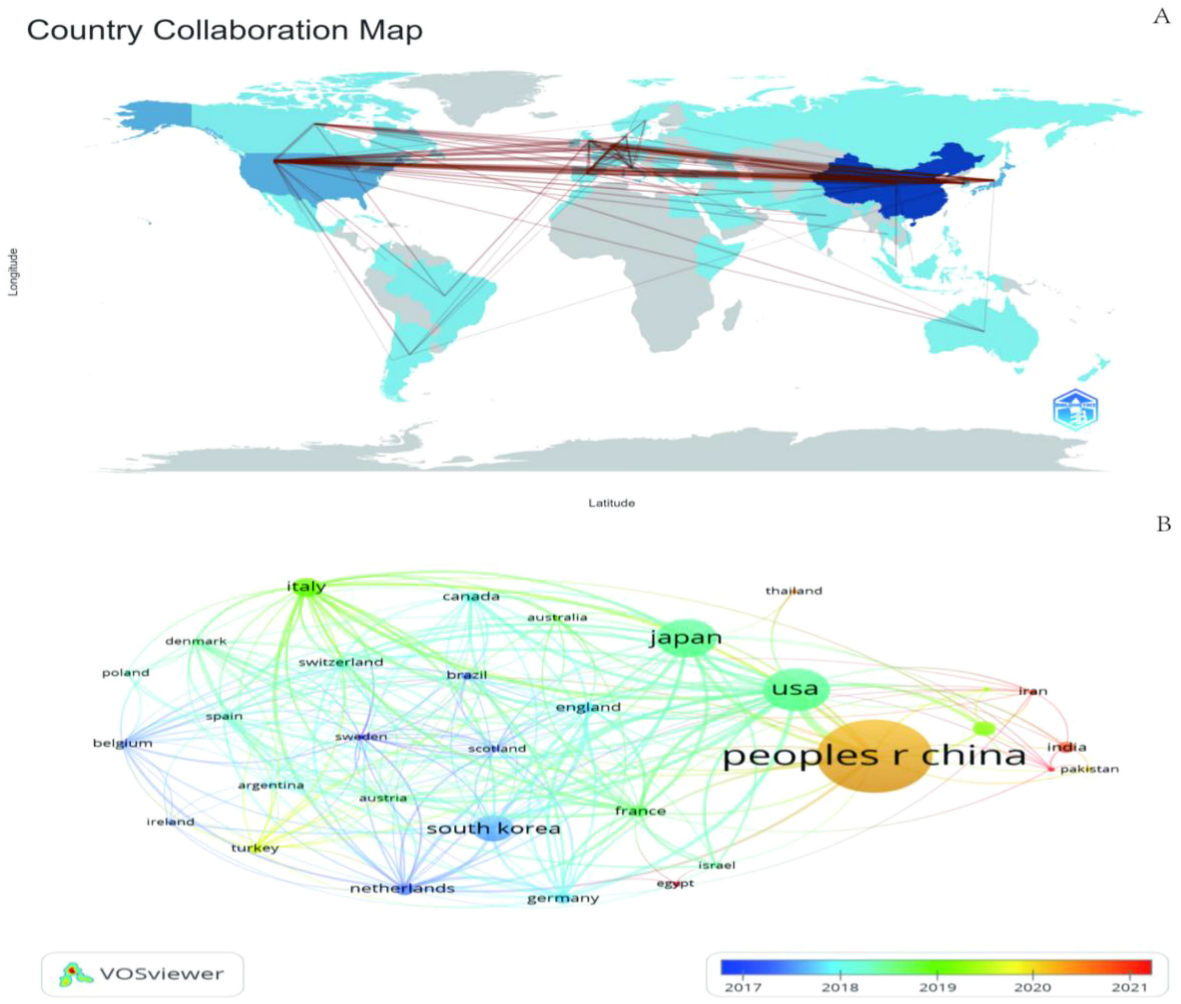
The geographical distribution **(A)** and visualization of countries **(B)** on research of GGNs.

We mapped the geographic distribution of countries associated with GGNs publications (**Figure 4A**), and the country co-occurrence mapping showed that these publications were distributed across 67 countries, with the top ten countries being mainly in Europe (n=5) (**Table 3**). The top three countries accounting for more than half of the total number of publications (n=1,758, 65.3%) were China in Asia (n=1,013, 45.7%), the United States in North America (n=401, 18.1%) and Japan in Asia (n=344, 15.5%).

**Table 3:** Top 10 countries and institution on GGNs publications.

Rank	Country	Counts	Rank	Institution	Counts
1	China	1,013	1	Shanghai Jiao Tong University	95
2	The United States	401	2	Fudan University	93
3	Japan	344	3	Seoul National University	84
4	Korea	195	4	Tongji University	59
5	Italy	113	5	Zhejiang University	44
6	Netherlands	60	6	Harvard Medical School	41
7	France	53	7	Sun Yat-sen University	39
8	Germany	51	8	National Cancer Center	37
9	India	51	9	Chinese Academy of Sciences	35
10	England	44	10	University of Texas MD Anderson Cancer Center	34

We filtered and visualized 32 countries based on the number of publications greater than or equal to 5 and constructed a collaborative network based on the number of publications and relationships in each country. There is much active collaboration between different countries. The collaboration network for each country based on the number of publications and relationships is shown in **Figure 4A**.

China has active collaborations with many countries including the United States, Japan, the United Kingdom, and France. Korea, on the other hand, has close ties with Japan, the UK, Italy, and the Netherlands. In addition, the project’s country colors represent the years with the highest number of publications from 2013 to 2023 (**Figure 4B**). For example, China’s yellow circle points out that the peak periods for research publication are late 2019 and early 2020.

### 3.4 Institutional analysis

**Figure 5:**
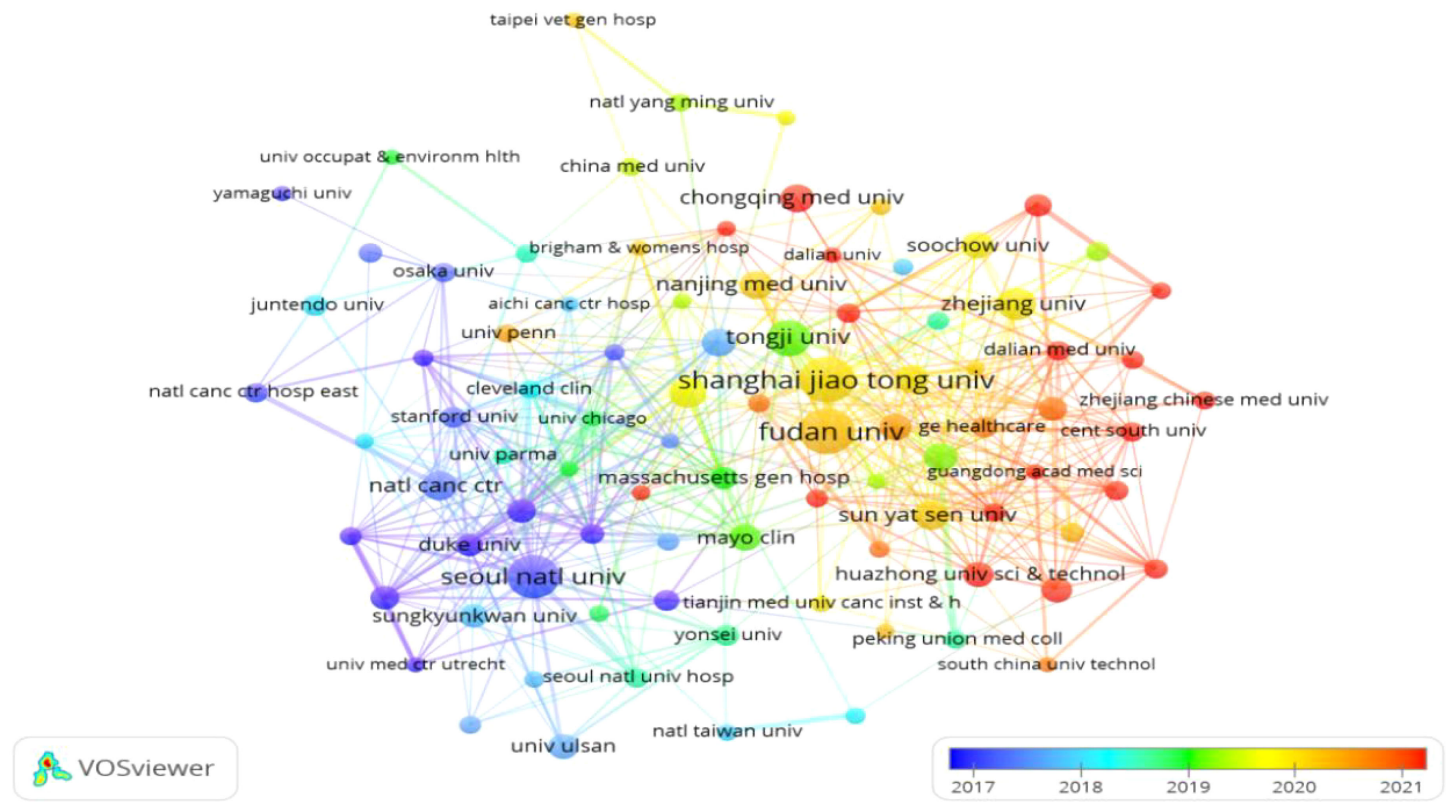
Collaborative mapping between 91 institutions with 10 or more publications.

Papers on GGNs-related research came from 2,274 institutions. We mapped the distribution of institutions with publications on GGNs based on publications greater than or equal to 5 or more (**Figure 5**), and the co-occurrence mapping shows that among the top 10 institutions in the field of pulmonary GGNs (**Table 3**), China has seven. The top three institutions are Shanghai Jiaotong University, Fudan University, and Seoul National University.

As can be seen in **Figure 5**, Shanghai Jiao Tong University, Fudan University, and Tongji University have very close collaborations, while Seoul National University has active collaborations with Anderson Cancer Center, Duke University, and Yonsei University.

### 3.5 Journals and co-cited journals

**Figure 6:**
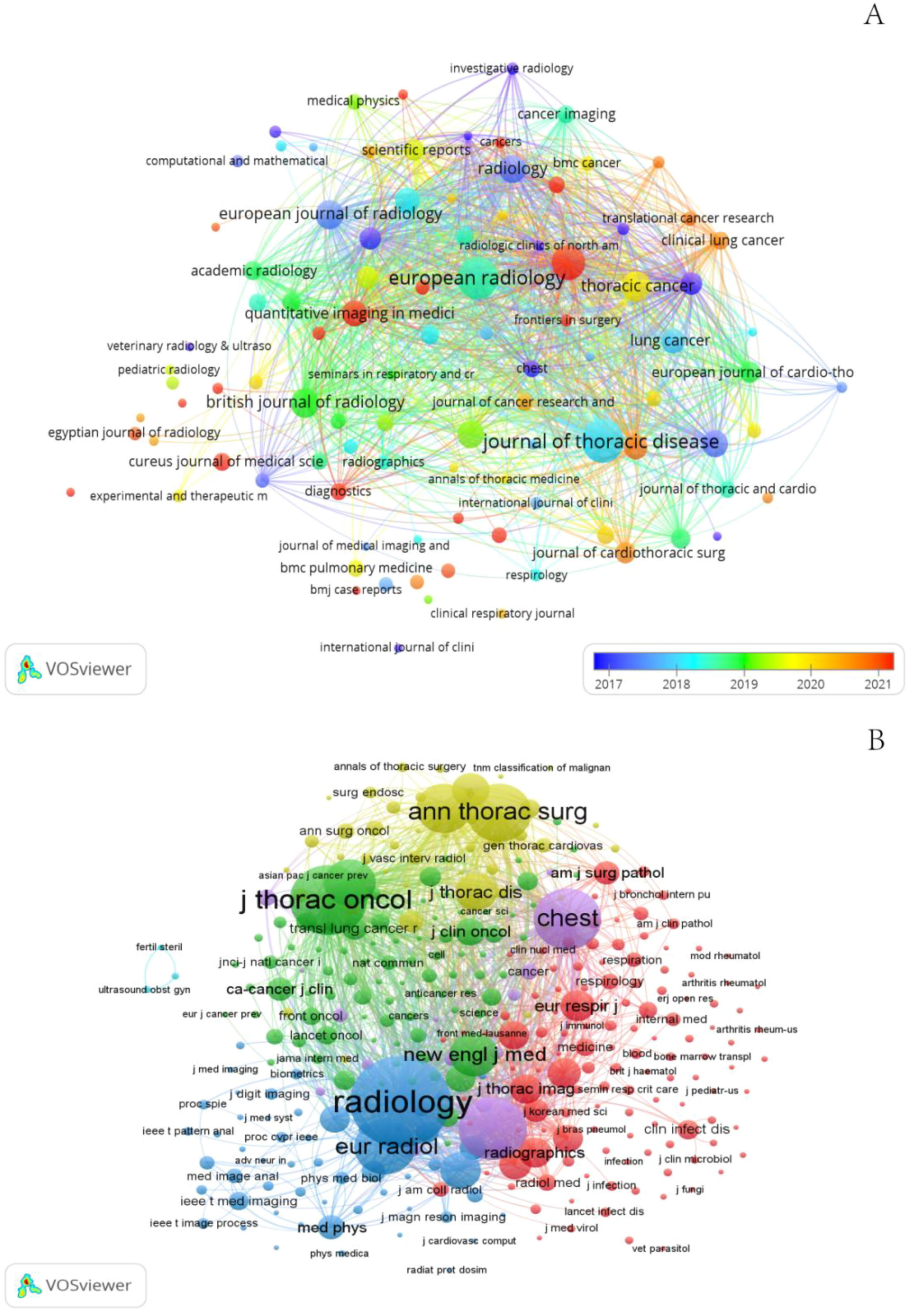
The visualization of journals **(A)** and co-cited journals **(B)** on the research of GGNs.

Publications related to GGNs were published in 498 journals. The largest number of publications was in the Journal of thoracic disease (n=100), Among the top 10 journals in terms of impact factor (**Table 2**), the highest impact factor was for Radiology (IF=19.7). Subsequently, we screened 98 journals based on the principle that the minimum number of relevant publications is equal to 5 and mapped the network of journals (**Figure 6A**). **Figure 6A** shows that European radiology has active citation relationships with journals such as Frontiers in Oncology, the Japanese Journal of Radiology, and the Journal of Thoracic Disease with Annals of Thoracic Surgery, Translational Lung Cancer Research, and Medicine.

**Table 2** shows that the top 10 active journals published a total of 552 papers, accounting for 24.89% of the total number of publications. Among the top 10 journals by publication volume, the highest impact factor was Radiology (IF=19.7), followed by the American journal of roentgenology (IF= 6.5) and European radiology (IF= 5.9). With four of the 10 journals in the Q1 (top 25% of the impact factor distribution), these findings indicate that articles on GGNs nodules are increasingly being published in high-level journals and that the high quality literature has greatly advanced research on this topic.

Journal co-citation analysis refers to the co-occurrence of 2 journals in the reference list of the third journal that publishes the cited literature, which allows for the location and classification of journals. We screened 335 journals based on the principle that the minimum number of relevant publications is equal to 20 and created a network map of co-cited journals (**Figure 6B**) and a table of the top 10 co-cited journals (**Table 2**). In addition, **Figure 6B** shows that Radiology has active citation relationships with journals such as the European Journal of Radiology, New England Journal of Medicine, and Clin Radio.

Among the top 10 co-cited journals, 70%(7/10) were from the United States, and the others were from Germany, the Netherlands and China. In the quartile category, seven journals were in Q1 in different areas.

### 3.6 Co-cited references

**Figure 7:**
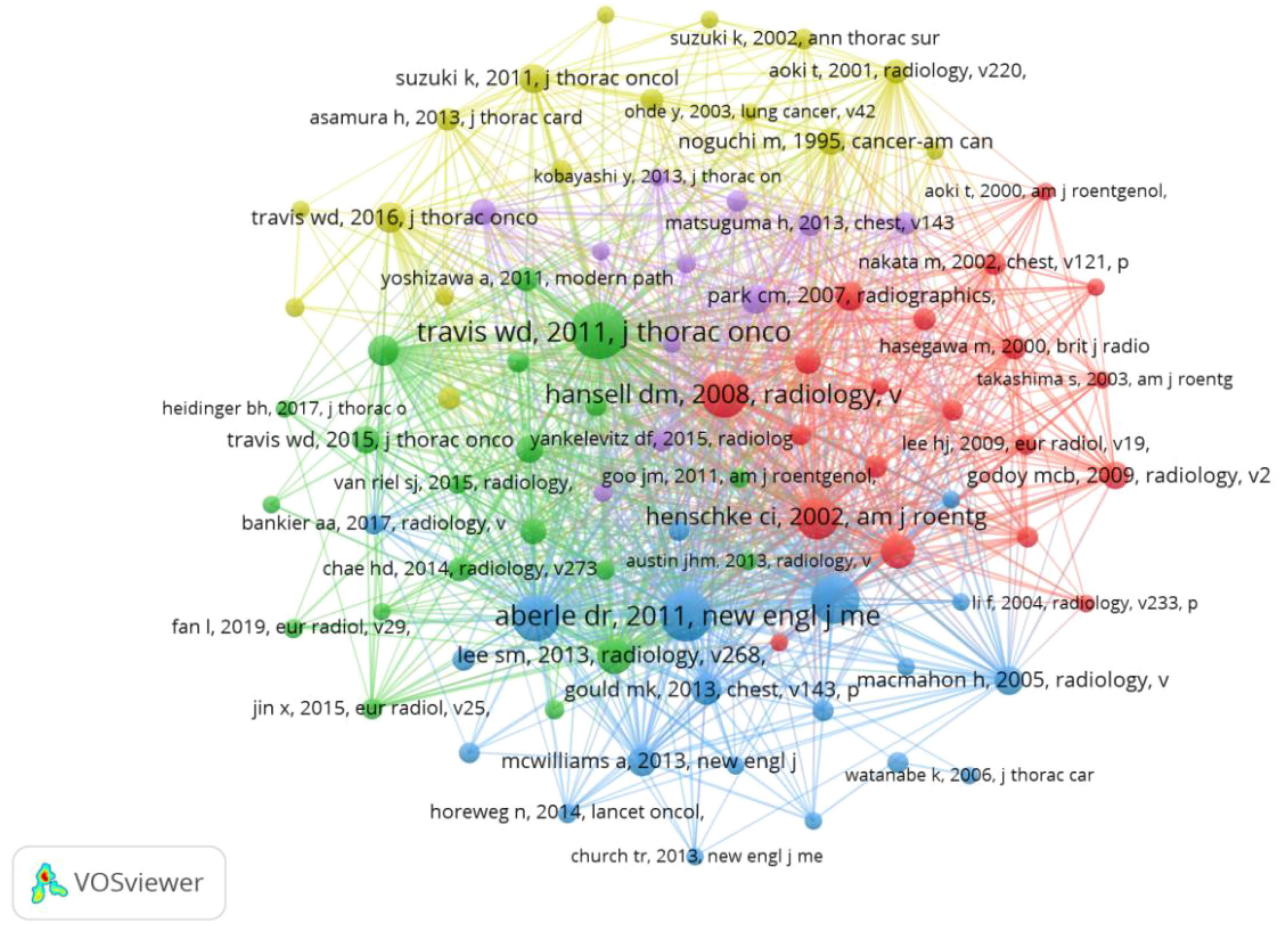
The visualization of co-cited references on the research of GGNs.

Co-Cited References refer to studies that have been co-cited by multiple other publications and can be considered as the research base of a field, and over the past decade, 31,411 studies on GGNs have been cited. After removing the relevant guidelines, the most cited article was “Reduced lung-cancer mortality with low-dose computed tomographic screening,” with more than 399 citations. **Table 4** describes the top 10 co-cited references and major studies. We selected the documents with 50 or more citations to construct a co-citation network diagram (**Figure 7**). From **Figure 6**, it can be seen that “naidich dp, 2013, radiology” has more than 399 citations compared with “kim hy, 2007, radiology”, “henschke ci, 2002, am j roentgen”, “kim hy, 2007, radiology”, and “henschke ci, 2002, am j roentgen”. 2002, am j roentgen,” and Aberle dr, 2011, New Engl j med has positive co-citation relationship with Lee sm, 2013, radiology and Maemahon h, 2017, radiology.

**Table 4:** Top 10 co-cited references on the research of GGNs.

Rank	Co-cited reference	Main research content	Citations
1	Travis WD, 2011, jthorac oncol, v6, p244 (15)	the International Association for the Study of Lung Cancer and others provide uniform terminology and diagnostic criteria for lung adenocarcinoma	486
2	Aberle dr, 2011, new engljmed, v365, p395 (16)	Screening with the use of low-dose CT reduces mortality from lung cancer.	399
3	Naidich dp, 2013, radiology, v266, p304 (17)	Six recommendations were made on subsolid pulmonary nodules	353
4	Hanselldm,2008, radiology, v246, p697 (18)	The Fleischner Society has updated a glossary of thoracic imaging terms that replaces a previously published glossary of thoracic radiography and CT scans	343
5	Macmahon h, 2017, radiology, v284, p228 (19)	The Fleischner Society guidelines for solid and subsolid nodules are combined into one table with specific recommendations for multiple nodules	342
6	Henschke ci, 2002, amjroentgenol, v178, p1053 (20)	In CT screening for lung cancer, the part-solid or nonsolid is more likely to be malignant than a solid one	268
7	Lee sm, 2013, radiology, v268, p265 (21)	In pure GGNs, a lesion size of less than 10 mm can be a very specific discriminator of preinvasive lesions from IPAs	195
8	Kim hy, 2007, radiology, v245, p267 (22)	To retrospectively compare pure pulmonary ground glass opacity (GGO) nodules observed on thin-section CT images with histopathologic findings	186
9	Travis wd, 2016, jthorac oncol, v11, p1204 (23)	Proposes codes for the primary tumor categories of AIS and MIA	154
10	Lim hi, 2013, chest, v144, p1291 (24)	Compare the morphologic features of persistent pure GGNs of ≥ 10 mm in diameter at TSCT scan with histopathology and patient prognosis	143

### 3.7 Reference with citation bursts

**Figure 8:**
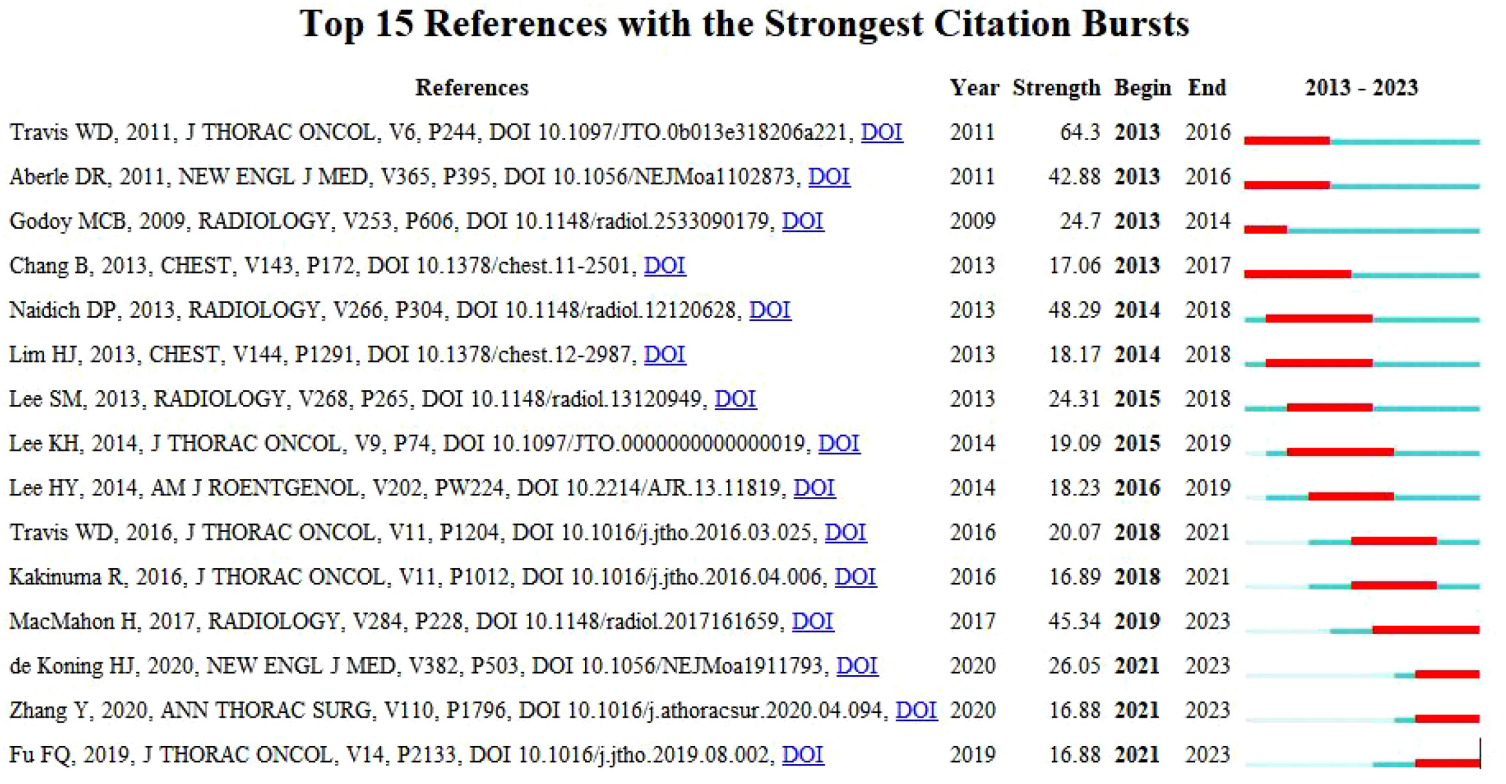
Top 15 references with strong citation bursts. A red bar indicates high citations in that year.

Reference with Citation Bursts is a document that is frequently cited by scholars in a particular field over a certain period. In our study, CiteSpace identified a total of 15 references with strong citation bursts (**Figure 8**), with bars indicating the year and red bars indicating the degree of strong citation bursts. **Table 5** describes the main research contents of the 15 references with strong citation bursts. The earliest citation outburst for a reference occurs in 2013, and the latest in 2021. The literature with the strongest citation burst (strength=64.3) was “International Association for the Study of Lung Cancer/American Thoracic Society/European Respiratory Society International Multidisciplinary Classification of Lung Adenocarcinoma” with citation outbreaks from 2013-2016. Overall, the outbreak intensity of the 15 publications ranged from 16.88 to 64.3, and the endurance intensity ranged from 1~ 4years.

**Table 5:** The main research contents of the 15 references with strong citation bursts.

Rank	Strength	Main research content
1	63.4	the International Association for the Study of Lung Cancer and others provide uniform terminology and diagnostic criteria for lung adenocarcinoma (15)
2	42.88	Screening with the use of low-dose CT reduces mortality from lung cancer (16)
3	24.7	Standardized management of subsolid nodules can be a precise means of predicting malignancy (25)
4	17.06	A strategy of long-term follow-up and selective surgery for growing nodules should be considered for pure GGO lung nodules (26)
5	48.29	Six recommendations were made on subsolid pulmonary nodules (17)
6	18.17	Compare the morphologic features of persistent pure GGNs of ≥10 mm in diameter at TSCT scan with histopathology and patient prognosis (24)
7	24.31	In pure GGNs, a lesion size of less than 10 mm can be a very specific discriminator of preinvasive lesions from IPAs (21)
8	19.09	Significant correlation between the size of the solid component on thin-section CT and the invasive component on pathology (27)
9	18.23	The histologic diagnosis of pure pulmonary GGNs, HRCT findings and pathologic correlation, and management (28)
10	20.07	Proposes codes for the primary tumor categories of AIS and MIA (23)
11	16.89	The frequencies and periods of development from PGGNs and HGGNs into part-solid nodules (29)
12	45.34	The Fleischner Society guidelines for solid and subsolid nodules are combined into one table with specific recommendations for multiple nodules (19)
13	26.05	Lung-cancer mortality was significantly lower among those who underwent volume CT screening than among those who underwent no screening (8)
14	16.88	The management of GGO-featured lung adenocarcinoma should be distinct from that of solid lesions (30)
15	16.88	Reveal the prognostic value of GGO components and differences in prognostic factors for part-solid and solid lesions in invasive stage I NSCLC (31)

### 3.8 Keywords analysis of research hotspots

**Figure 9:**
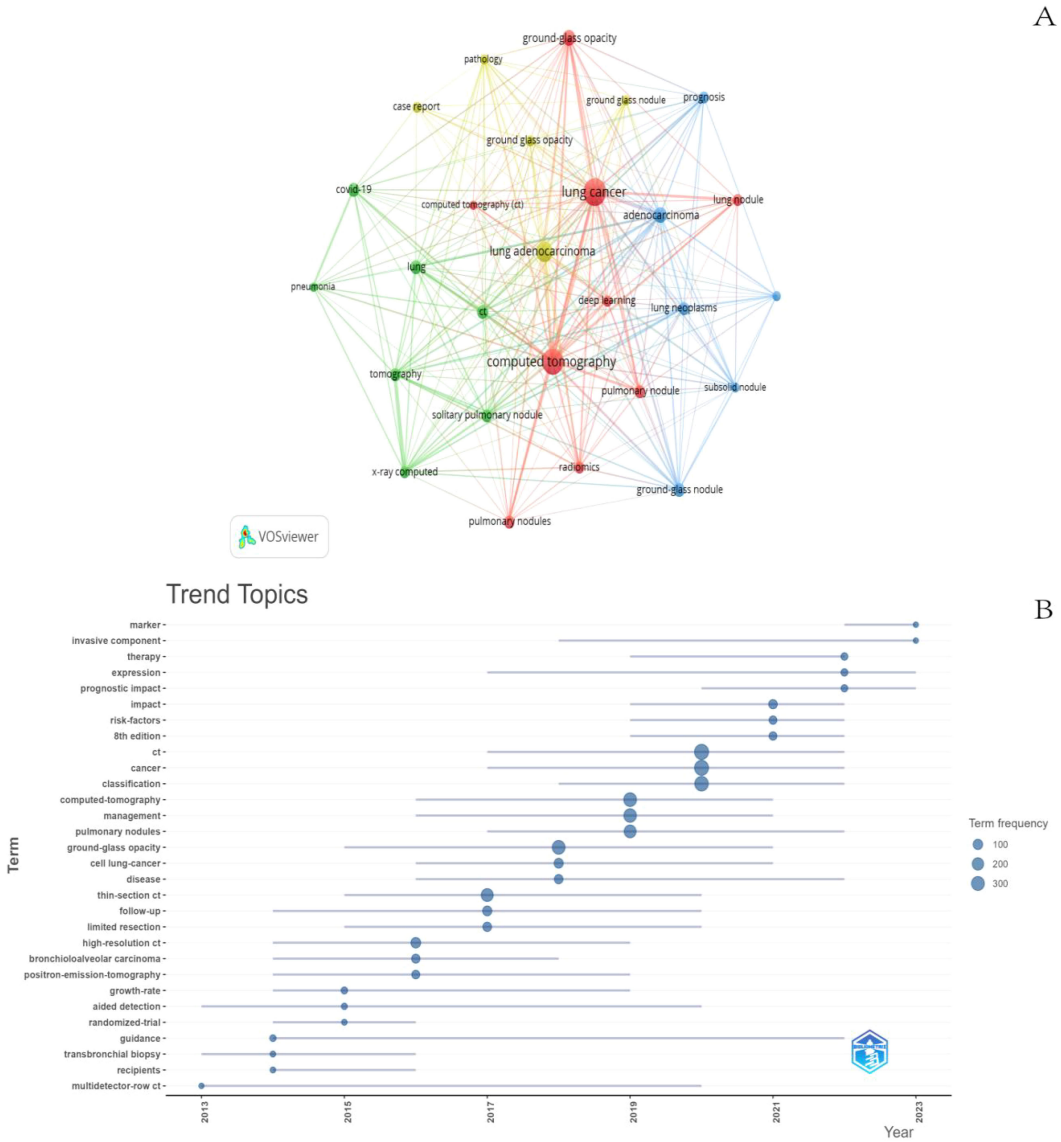
Keyword cluster analysis **(A)** and trend topic analysis **(B)**.

The co-occurring keywords reflect the research focus in the field of GGNs, and through the co-occurrence analysis of keywords, the research hotspots in a certain field can be quickly captured. The top 10 keywords in this field include “CT, lung cancer, lung adenocarcinoma, adenocarcinoma, lung nodule, ground glass component, covid19, isolated lung nodule, imaging histology, diagnosis”. Among them, “lung cancer” and “CT” appeared more than 200 times, representing the main research direction of GGNs. We filtered the keywords with the number of occurrences greater than or equal to 40 and performed cluster analysis by VOSviewer (**Figure 9A**), which resulted in a total of 27 keywords, with a total of 4 major categories. The shorter the line between nodes, the stronger the connection between keywords. As shown in **Figure 8A**, we obtained four clusters in total. The keywords in green clusters consist of COVID-19, lung, pneumonia, tomography, x-ray computed, and solitary. The keywords in green clusters consist of COVID-19, lung, pneumonia, tomography, x-ray computed, solitary, pulmonary nodule, ct, etc. The keywords in red clusters consist of lung cancer, GGO, deep learning, radiomics, CT, pulmonary nodules, etc. The keywords in red clusters consist of lung cancer, ground-glass opacity, deep learning, radiomics, CT, pulmonary nodules, etc. The keywords in blue clusters consist of prognosis, adenocarcinoma, subsolid nodule, etc. The keywords in yellow clusters consist of pathology, case report, lung adenocarcinoma, etc. We can observe that “lung cancer” is closely related to other keywords.

The analysis of keyword trend themes (**Figure 9B**) shows that the research in the period of 2013-2023 mainly focuses on the classification, identification, and management of GGNs. The main keywords are CT, Lung Cancer, and Deep Learning. In recent years, three keywords: marker, invasive component, and therapy can be seen to appear frequently in the last two years, most likely representing the current research hotspots.

## 4 Discussion

### 4.1 Global trends in the investigation of GGNs.

Understanding the latest research findings is increasingly challenging. To present the current global scientific output on GGNs, we conducted a bibliometric analysis using 10 years of data from 2013 to 2023. This approach provides a novel technique for managing and illustrating the knowledge structure of specific studies.

The increasing detection rate of GGNs has brought a series of medical problems. Correct identification and treatment of GGNS are of great significance for the prevention and treatment of lung cancer. The related published literature has also been increasing year by year in the past decade, and **Figure 2** shows the huge growth of global publications on ground glass lung nodules research in the past 10 years. The annual number of published papers in 2013 was 80, indicating that relevant scholars had paid attention to GGNs ten years ago and carried out relevant research, and the corresponding research foundation had been established. From 2013 to 2023, research in this field has progressed rapidly. Since 2014, more than 100 papers have been published every year, which indicates that the research is in an explosive period and related research has attracted the attention of more and more scholars. Among the top ten most prolific authors, Jin Mo Goo published the most papers and took a major leadership role in the field. Therefore, the authors and their team are more likely to important GGNs related papers published. He has focused on the value of chest imaging for early lung cancer screening. As early as 2011, Jin Mo Goo suggested in his paper “Ground-glass nodules on chest CT as imaging biomarkers in the treatment of lung adenocarcinoma” that GGNs could be used as imaging markers for lung adenocarcinoma and that their progression reflected the disease process of lung adenocarcinoma (32). In terms of co-cited authors, Travis WD (citation=960) was the most cited author, followed by Henschke CI (citation=610) and Suzuki K (citation=499). In 2011, Travis WD (15) published the most co-cited study, which initially summarized and introduced the novel concept of precancerous stages of GGNs, including adenocarcinoma *in situ*. As early as 2002, Henschke CI (20) published a multicenter study demonstrating that annual spiral CT screening markedly increased the detection rate of lung cancer in comparison to conventional X-ray, thereby reducing the risk of mortality from lung cancer. This established the importance of CT screening, and a series of studies were subsequently conducted on CT-detected GGNs. The results of these two studies constituted a significant advance in the study of GGNs.

A survey of countries and institutions revealed that China and the United States have a significantly larger number of publications than other countries and regions. They are also the most prolific in this field. However, a review of the relevant literature indicates that although Chinese research institutions have published a considerable number of articles, they require both innovative and classical literature. Consequently, the influence of Chinese research institutions in the international arena has yet to be strengthened. The United States continues to lead the field, with the highest number of citations and higher impact articles and therefore performs better in terms of the level of research. In addition, through the analysis of publications, we can see that differences in nodule characteristics between racial groups may prompt researchers to pursue more in-depth studies in different regions, resulting in differences in the number of publications. We found that the distribution of GGNs related publications is dominated by countries in East Asia, with Japan and South Korea together publishing more publications than the United States. The dominance of East Asian countries in GGNs related publications, especially China, Japan, and South Korea, shows the active research in this field in this region. This may be related to the specific focus on early screening and diagnosis of lung cancer in these countries, and also reflects the gradually in-depth study of nodule characteristics and their prognosis in this region. Given previous studies, racial differences in lung cancer prevalence and differences in the characteristics and prognosis of GGNs presenting as early-stage lung cancer between Asians and Caucasians will undoubtedly influence the direction of research and the presentation of results. Most randomized controlled trials of lung cancer screening in Europe and the United States have only been conducted in smokers or recent quitters. The incidence of lung cancer in non-smoking patients is increasing year by year, especially in Asian female patients, which is mainly manifested as early stage lung adenocarcinoma GGNs (33).

There are also disparities in the gene mutation spectrum between Asians and Caucasians. In Asia, the mutation rate of EGFR amounts to 40%-55%, whereas it merely constitutes 15%-25% in Caucasians (34). Therefore, differences in genetic background also lead to differences in the incidence of lung cancer in non-smoking patients, and also promote the study of GGNs in East Asian countries.

In terms of nodule risk prediction, Mayo’s nodule risk prediction model is currently the main risk assessment tool for nodules, but it is mainly based on the data of white population, so it may not fully consider the specific risk factors and nodule characteristics existing in the Asian population. For example, nodules in the upper lobe of the lung are not suitable for Asians as high-risk factors. East Asian patients with pulmonary tuberculosis are also more likely to present with upper lobe location and do not have the same prognostic risk, which may further complicate the characteristics and risk assessment of nodules. Therefore, the diversity of nodule characteristics in different racial backgrounds may prompt researchers to investigate these differences more deeply, which may lead to differences in the number of publications. This also reflects the need to adjust and optimize according to the characteristics of different populations when developing clinical guidelines and rheumatism risk assessment models. Researchers need to pay more attention to these differences in order to provide a more targeted framework for clinical practice. The ACCP Asian Clinical Practice consensus guidelines are developed to reflect the characteristics of East Asian population, and emphasize the importance of monitoring GGNs of all sizes (35). This recommendation takes into account the increased risk of malignancy in this population, indicating that the management of GGNs needs to be individualized in combination with specific population characteristics. Seven of the top ten institutions are from China. A review of the literature reveals that the papers published by these structures have received substantial grant funding, which reflects the importance that the government places on the field. This explains why China is involved in most of the papers on research on GGNs. These findings also suggest that the establishment of top-notch research institutions is the key to improving the status of a country’s academic community.

Concerning the establishment of collaborative relationships between research institutions, a number of institutions have successfully forged productive partnerships. For instance, there is a particularly close relationship between Shanghai Jiao Tong University, Fudan University, and Tongji University. Seoul National University has established active collaborative relationships with the Anderson Cancer Center, Duke University, and Yonsei University. The evidence above indicates that numerous stable research teams have been established in GGNs-related research. However, these teams are primarily situated within the confines of their respective countries. While collaborative relationships exist between some countries, there is considerable scope for strengthening the breadth and intensity of inter-institutional cooperation. For instance, inter-institutional collaboration between individual countries is constrained, and the necessity for enhanced international collaboration is apparent. This situation will undoubtedly impede the long-term development of the research field. Consequently, extensive cooperation and exchanges between research institutions in various countries will be conducive to jointly overcoming technical barriers and comprehensively assessing and classifying high-risk GGNs populations for precise prevention and treatment. However, it is worth noting that uniform screening standards, such as standard imaging equipment and nodule diameter measurements, should first be applied before formalizing the cooperation. This is because different screening tools are a confounding factor in study results. Furthermore, among the most highly ranked journals in terms of relevant GGNs publications, the Journal of *Thoracic Disease*, *European Radiology*, and *Frontiers in Oncology* are likely to be the principal journals in which articles of GGNs are published. It may be advisable to submit additional articles to these publications. However, among the top ten journals in terms of publication volume, only one journal had an IF higher than 10.0, which indicates that the currently published articles have a limited impact and that there is a lack of classical literature.

Among the co-cited journals, we can find a high number of core authoritative journals in the field of GGNs. Most of them are high-impact Q1 journals, the top two journals being *Radiology* (6,278 co-citations) and *Journal of Thoracic Oncology* (3,657 co-citations). Two sets of results were found, suggesting that relevant research is published mainly in two types of journals: medical/medical/clinical and radiology/physics. This suggests that GGNs are becoming recognized as a problem in the clinic, and relevant research is being carried out. The focus of research has been mainly on chest radiology, especially the use of CT in the diagnosis of benign and malignant GGNs and the prediction of invasiveness. In addition, the number of related basic research journals is gradually increasing, indicating that scholars have begun to explore the genetic and molecular mechanisms of the onset and development of GGNs, to elucidate the underlying causes of the occurrence and development of GGNs from a microscopic perspective.

Co-cited reference analysis results show that Travis WD (15) published the most cited study in 2011, introducing new concepts of adenocarcinoma *in situ* (AIS) and minimally invasive adenocarcinoma (MIA). It laid the groundwork for subsequent research into the pathology of GGNs. Of the ten total cited papers, Professor Travis, WD has published a total of two papers in 2016, in addition to the initial review, which further summarized the recommendations for coding the primary tumor categories of AIS and MIA for the forthcoming 8th edition of the TNM lung cancer classification will include the coding of the T-category of sub-solid nodules and the assessment of tumor size in some solid tumors. This will provide a uniform method of measurement and a specific means of assessing GGNs, which is an important clinical guideline (23). Also in 2011, Denise R Aberle published “Reduced lung-cancer mortality with low-dose computed tomographic screening” in *the New England Journal of Medicine*. The study found that low-dose CT screening reduced lung cancer mortality by only 20% compared with X-ray screening. This clinical trial established the importance of low-dose CT in lung cancer screening and provided the scientific basis for the rapid development of CT in screening (16). In 2013, David Naiditsch published “Recommendations for the management of subsolid pulmonary nodules detected on CT: a statement from the Fleischner Society”. This document outlines a standardized approach to the interpretation and management of subsolid pulmonary nodules. It includes six constructive comments, clarifies strategies for the duration of follow-up for different classifications of GGNs and identifies future directions (17). In summary, the top 10 co-cited papers primarily focused on the imaging clinical features of GGNs. They developed benign and malignant differential diagnoses and follow-up strategies, initially discovering the progression pattern of GGNs based on imaging in the clinic and managing it scientifically. Among the highly cited papers, the main institutions are still based in the United States, which further confirms the dominant position of the United States in GGNs-related research.

### 4.2 Hot topics and frontiers

“Reference with citation bursts” represents emerging themes in specific research areas, as evidenced by their high frequency of citation by researchers in recent years. The study of the imaging characteristics of GGNs, as well as the identification and management of benign and malignant tumors, have remained the main themes of the decade, according to the main research content of the highly cited references. In addition to the exponential growth in the number of citations, keywords also facilitate the rapid identification of research hotspots in the field of GGNs. The most prominent keywords in this context encompass Lung Cancer, CT, and Deep Learning, which collectively reflect the principal research areas currently driving the investigation of ground-glass lung nodules.

#### 4.2.1 GGNs and lung cancer

The rising incidence and high mortality rate of lung cancer underscore the necessity for lung cancer prevention and treatment, and prompt diagnosis and treatment have emerged as a crucial strategy to reduce lung cancer mortality. In clinical practice, it has been observed that the persistence of GGNs may be the main sign of early lung adenocarcinoma. However, early lung cancers with GGNs show “inert” progression, with a volume doubling the time between 769 and 1,005 days, and rarely metastasize, with a good prognosis (36). Consequently, as a distinct category of early-stage lung cancer, the investigation of the underlying mechanism responsible for the inert progression of GGNs has emerged as a pivotal research area for scholars. An increasing number of scholars have employed second-generation sequencing techniques to elucidate the molecular mechanisms governing the inert progression of GGNs. The majority of scholars focus their research on the gene mutation characteristics and the immune microenvironment, according to the different pathological stages of GGNs. For instance, Zhang (37) discovered that the mutation burden and copy number of GGNs gradually declined with the development of nodal pathology, indicating a possible association between these characteristics and nodal pathological progression. Single-cell sequencing revealed that early-stage lung cancers presenting as ground-glass lung nodules have a less active metabolic and immune microenvironment, with lower relative abundance of myeloid and NK cells than solid nodules (38). This suggests that GGNs may have a lower tumor load and therefore less tumor-associated antigen release, which is not recognized by immune cells and there is less immune escape. This may be a potential molecular mechanism for their slow clinical progression. It is important to note, however, that GGNs are highly heterogeneous. While inert GGNs are prevalent, some nodules are still found to have malignant potential for rapid progression or even metastasis in clinical findings. Further exploration of the characteristics of early-stage lung cancers exhibiting GGNs from a molecular point of view would therefore help differentiate between GGNs with different growth potentials for early prevention.

#### 4.2.2 GGNs and CT

The widespread use of low-dose spiral CT in lung cancer screening has led to the identification of a large number of GGNs (16). The diagnosis and management of these nodules initially centered on CT imaging, with density becoming the primary focus of investigation. In GGNs presenting as early-stage lung cancer, there is a correlation between the proportion of ground-glass density and patient prognosis and survival. Previous studies have corroborated the assertion that a reduction in the proportion of ground glass and an increase in solid components within lung nodules are independent risk factors for lymph node metastasis (39). Furthermore, research has indicated that an elevated ratio of solid components is linked to an enhanced invasive capacity of lung cancer (40). It is important to note that even lung cancers with a minimal ground glass component have a more favorable prognosis than pure solid lung cancers. However, there is a lack of consensus among studies regarding the relationship between the percentage of reduction in the ground glass component and the degree of malignancy. In addition to density, several studies have demonstrated that nodule diameter, volume, and imaging features such as lobulation, burr, and vascular penetration are risk factors for nodule malignancy (41). However, it is important to note that even if a GGN is malignant, a short-term observation strategy does not significantly impact surgical strategy or the patient’s survival prognosis. The current standard of care for GGNs is regular follow-up for varying durations, based on the imaging characteristics of the nodule and the degree of risk. This approach has been recommended in guidelines in various countries. Although early screening for lung cancer can reduce lung cancer mortality, there are still some potential harms associated with screening for lung nodules. Studies have shown that the widespread use of LDCT screening can lead to overdiagnosis of up to 18.5% of patients and surgical interventions in 0.5% to 1.3% of screened patients, resulting in a waste of healthcare resources (42, 43).

#### 4.2.3 GGNs and deep learning

The development of artificial intelligence technology in the field of medicine is progressing at a considerable rate. Deep learning, which represents a pivotal technology of artificial intelligence, is assisting medical professionals in the formulation of rapid and objective clinical judgements and the enhancement of their efficiency. Deep learning is a novel machine learning method based on big data that employs parallel and distributed computing, as well as intelligent computing. It has its roots in the study of artificial neural networks, which emulate the computational model of the human brain. Through a process of continuous learning and parameter adjustment, the network is able to approximate any complex function (44).

Considering the considerable number of heterogeneous ground-glass lung nodules that have been identified, it is crucial to implement non-invasive techniques for the precise diagnosis and accurate pathological identification of GGNs. The utilization of automated tools may potentially alleviate the healthcare burden associated with this workflow. Furthermore, Deep Learning has the potential to efficiently extract the imaging features of the nodules and combine them with other relevant clinical information of the disease, thereby enabling the construction of a combined model that can accurately predict the benignness, malignancy, aggressiveness, and prognosis of GGNs (45–49). This approach has the potential to significantly enhance diagnostic accuracy.

Convolutional neural networks (CNNs) represent the most prevalent neural network structure employed for deep learning applications in vision. They are capable of accurately detecting and segmenting GGNs by integrating images from CT scans. This enables the automatic calculation of various parameters, including diameter, density, volume, mass, volume doubling time (VDT), and mass doubling time (MDT). Additionally, CNNs can integrate patient-specific clinical characteristics and potential test indicators obtained from clinicians to develop more efficient training techniques and novel models. Deep Learning is a widely used technique in lung cancer screening, offering a potential solution to the limitations of human error and improving overall efficiency. A number of studies have demonstrated that Deep Learning exhibits a high degree of concordance with radiologists in the assessment of nodule measurements, diagnosis, and prognosis, and holds considerable promise for the future (50, 51).

Pan (52) developed six ternary models for classifying pre-invasive, minimally invasive, and invasive adenocarcinomas. This was achieved by combining framework optimization, joint learning, and adjudication methods. The objective was to optimize deep learning models for classifying invasive adenocarcinomas of GGNs. Additionally, the aim was to improve their diagnostic performance. Qi (53) developed a three-dimensional automated deep learning model, Lung-PNet, which performs automatic segmentation and classification of GGNs. The model could classify GGNs in different pathological stages and help to guide the management of GGNs. Tang (54) developed four deep-learning models to predict the growth of GGNs and found that the DenseNet_DR model had a superior predictive performance, which could assist doctors in predicting the growth of GGNs. The utilization of multimodal data for computation represents a key advantage of deep learning. Zhang (55) developed a multimodal prediction model comprising plasma DNA methylation markers and CT shadowgraph images, intending to distinguish early-stage lung cancers from benign GGNs. This was achieved through a deep learning approach, which could potentially facilitate the classification of patients into good or poor prognosis groups based on the prognostic indices of DNA methylation and lung cancer driver gene alterations.

However, the models related to various GGNs based on deep learning are limited by a small clinical sample size, the majority of which are single-center retrospective studies, and a weak level of evidence. These limitations require validation by larger datasets and more rigorous validation. As scholars continue to investigate GGNs and the technology continues to evolve, a growing number of potential markers combined with deep learning are demonstrating significant potential for clinical application and warrant further investigation.

#### 4.2.4 Marker of GGNs

Given the considerable heterogeneity of GGNs, the identification of their benign or malignant nature is of paramount importance for subsequent treatment management. However, due to the small size of GGNs and their location in the periphery of the lungs, it is challenging to ascertain the pathological nature of GGNs through biopsy via bronchoscopy or puncture. Consequently, the popularity of low-dose helical CT has led to the detection of a significant number of GGNs, prompting the investigation of the prediction of benignity and malignancy of GGNs based on the characteristics of CT imaging. As early as 1997, the Mayo Clinic’s model for predicting the benign and malignant risk of lung nodules suggested that the diameter, presence of burrs, and location of nodules on CT imaging should be included as important elements of the risk of malignancy (56). As the research continues, more and more scholars have converted the visual CT imaging features into more quantifiable data for analysis. Many of the imaging features have been confirmed to be associated with the benign and malignant risk of GGNs. The imaging features of GGNs, including CT Value(CT Value = (RawPixelValue * RescaleSlope) + Rescale Intercept, A unit of measurement that determines the density of a local tissue or organ in the human body), VDT, percentage of solid components, surface-to-volume ratio, and others, are predictive of GGNs-related imaging genomics (57). The prediction model is constructed based on a large number of imaging features, combined with clinical information and genomic data. The mined data features have the potential to judge and assess tumor heterogeneity more effectively than other data sources. The researchers extracted imaging features, including the diameter and location of GGNs in CT scans, and established a joint model for the identification of benign and malignant GGNs. This model demonstrated superior discriminatory performance and was able to distinguish between benign and malignant GGNs with greater accuracy, offering significant clinical value (58, 59). Some researchers have also used the entropy value in imaging histology to distinguish benign and malignant GGNs (60).

Furthermore, the researchers discovered that both the tumor microenvironment and the peritumor microenvironment play a role in the development and progression of early-stage lung cancer (61). To enhance the accuracy of benign and malignant GGNs predictions, researchers extracted intratumorally and peritumoral imaging and histological features of GGNs and employed these features to construct a combined intratumorally and peritumoral imaging and histological model, which exhibited a high degree of predictive efficacy (62, 63).

As the efficacy of detection methods continues to improve, early lung cancer diagnostic models that combine liquid biomarkers with clinical imaging features often demonstrate greater sensitivity. The advent of liquid biopsy as a non-invasive tool has opened up a new avenue for the early diagnosis of lung cancer. This is an area of great interest, given the involvement of various oncogenes, oncogene signaling pathway components, and other cellular processes in the molecular pathogenesis of lung cancer. Imaging histologies commonly combined with liquid biopsies include MicroRNAs(miRNAs), long-stranded non-coding RNAs, DNA methylation, circulating tumor DNA, circulating tumor cells, and lung cancer-associated proteins. In particular, the combined diagnosis of liquid biopsy and low-dose CT may be a promising area of research for the early diagnosis of lung cancer in recent years. To identify the benign and malignant nature of GGNs, Ye (64) developed a diagnostic model for early lung cancer by combining imaging histological features with liquid biopsy in 560 patients with GGNs. The model was subsequently validated in an independent cohort with an AUC of 0.895, which was higher than the Mayo Clinic model and the VA model. He (65) screened a total of 11 variables by combining imaging and histological features of lung nodules in conjunction with serum fluid markers (CEA, NSE). Then, it aggregated them to generate a new predictive model (AUC of 0.768), which showed better screening performance. Zhang (55) used DNA methylation levels, lung nodule clinical phenotypes, and LDCT in a population of 257 cases to jointly build a multimodal predictive model, which outperformed the Mayo Clinic model in an independent validation set and found that patients with SHOX2/PTGER4 DNA hypermethylation were enriched with TP53 mutations, suggesting that DNA methylation levels and lung cancer driver gene alterations can be used as prognostic indicators of benign and malignant. MiRNA is a non-coding, short, single-stranded RNA with an average size of 22 nucleotides, which is involved in the pathophysiological processes of the organism by regulating gene transcription and translation (66). MiRNA as a biomarker to judge the nature of pulmonary nodules has also been recently studied. Recent studies have shown that the sensitivity of miRNA as a biomarker to differentiate malignant pulmonary nodules ranged from 54.8% to 92.9%, and the specificity was 69.2% to 90.9% (67). Previous studies have shown that single miRNAs are of low diagnostic value for lung nodules and need to be combined with multiple miRNAs and other clinical features to obtain better diagnostic results (68). Relevant studies show that miR-146a, miR-200b, and miR-7 may be biomarkers for early lung cancer in patients with lung cancer (69).

In addition, with the continuous advancement of various genomic technologies such as genomics, proteomics, metabolomics, and volatomics, as well as the development of various disciplines such as molecular biology, detection technologies, and statistics, coupled with the application of emerging technologies in the field of medical diagnosis and treatment such as artificial intelligence, Internet of Things and robotics, the diagnosis of early-stage lung cancer has made great progress. The relevant non-invasive biomarkers to predict benign, malignant, and invasive GGNs are becoming more common. Sani’s team analyzed fasting and post-prandial breath samples from early-stage lung cancer patients compared to healthy controls at pre-operative, 1-hour post-operative, 3 to 7 days post-operative, and 4 to 6 weeks post-operative. The results of the study showed that in early-stage lung cancer patients 3-hydroxy-2-butanone (TG-4), ethanol aldehyde (TG-7), 2-pentanone (TG-8), acrolein (TG-11), nonanal (TG-19), decanal (TG-20) and crotonaldehyde (TG-22) were significantly different from healthy controls within 1 hour after a meal. This non-invasive methodological study of volatile organic compounds (VOCs) as novel cancer biomarkers identified exhaled compounds associated with early-stage lung cancer. It provides new ideas for early screening of lung cancer markers (70). In addition, there are other avenues of research, such as lncRNA, cfDNA, and specific flora in patients’ saliva, which have received attention in the current study (71–73). Relevant biomarkers are also involved in multiple dimensions of GGNs, including prediction of GGNs growth trend, prediction of GGNs pathology-related indices, prediction of GGNs mutations and immunotherapy-related markers, and so on (32). Through the integration of various technological tools and interdisciplinary collaboration, we expect to achieve early detection and precise treatment of lung cancer, thus improving the survival rate and quality of life of patients.

## 5 Advantages and shortcomings

This study has several unique strengths. Firstly, for the first time, we employed bibliometric methods to systematically analyze the last ten years of research related to GGNs. This article can provide a comprehensive guide for scholars concerned with related research. Secondly, we investigated the use of three bibliometric tools simultaneously, two of which (VOSviewer and CiteSpace) have been widely used in the field of bibliometrics. Therefore, our data analysis process is likely to be objective. Finally, bibliometric analyses offer a more comprehensive understanding of emerging trends and cutting-edge issues than traditional reviews.

This study also has some things that could be improved. Firstly, the data in this study came only from the WoSCC database, ignoring other databases, which may have missed some relevant studies. Second, we filtered studies published in English, which may mean that non-English writing papers were underestimated. Finally, as research in the field continues to develop and expand, future studies should endeavor to incorporate the latest findings, which could provide valuable information and guidance for continued research and development in the field.

## 6 Conclusion

GGNs have important research value and application prospects in the diagnosis and treatment of early-stage lung cancer. The rapidly increasing number of publications indicates that research on GGNs is attracting more and more attention from scientists around the world. The leading country is still the United States, but China is developing rapidly; cooperation and communication between countries and institutions need to be strengthened. Relevant non-invasive biomarkers of GGNs have been the focus of research in recent years and have great value for clinical application. In addition to relevant clinical research, basic research on GGNs is gradually being carried out based on the rapid development of multi-omics technology. Representative single-cell sequencing technology has also revealed the unique tumor microenvironment of GGNs, which is changing from vague knowledge to the search for more appropriate means of intervention for GGNs.

## Data Availability

Publicly available datasets were analyzed in this study. This data can be found here: pubmed.
